# Development and validation of a predictive model for early blastocyst formation on day 4 post-fertilization

**DOI:** 10.3389/fendo.2026.1752963

**Published:** 2026-04-01

**Authors:** Yasong Geng, Fangfang Dai, Haoyang Dai, Linlin Tao, Jing Ma, Jiayi Yin, Bo Zheng

**Affiliations:** 1 Xingtai Key Laboratory of Reproductive Medicine, Xingtai Meihe Reproductive and Genetic Hospital, Xingtai, China; 2 Hebei Key Laboratory of Reproductive Medicine, Hebei Reproductive Health Hospital, Shijiazhuang, Hebei, China; 3 Institute of Biology, Hebei Academy of Sciences, Shijiazhuang, Hebei, China

**Keywords:** artificial intelligence, early blastocyst, *in vitro* fertilization-embryo transfer, ongoing pregnancy, selective single embryo transfer

## Abstract

**Objective:**

To develop and validate a predictive model for early blastocyst formation on Day 4 post-fertilization using clinically accessible parameters.

**Methods:**

This retrospective cohort of 2,557 patients scheduled for fresh Day 4 embryo transfer was randomly divided into training (n=1,789) and validation (n=768) sets at Xingtai MeiHe Reproductive and Genetic Hospital from January 2021 to December 2025. Early blastocyst formation occurred in 44.9% (training) and 44.1% (validation) of cases. Multivariable logistic regression and nomogram modeling were employed, with model performance assessed via AUC, calibration curves, and decision curve analysis.

**Results:**

After multivariable adjustment, the number of Day 3 embryos with >10 blastomeres (aOR=1.668, 95% CI: 1.374~2.025, P<0.001) and those with iDAScore >4.8 (aOR=1.369, 95% CI: 1.190 ~ 1.575, P<0.001) were the only independent predictors of Day 4 early blastocyst formation, while 15 covariates including ovarian reserve markers and sperm parameters showed no significant association (P>0.05). The nomogram integrating Day 3 embryos with >10 blastomeres and iDAScore>4.8 demonstrated robust discrimination (training AUC = 0.782, 95% CI: 0.738 ~ 0.826; validation AUC = 0.773, 95% CI: 0.705 ~ 0.841) with sensitivity of 65.4~65.9% and specificity of 79.6 ~ 81.7%. Calibration curves indicated minimal prediction deviation, and decision curve analysis confirmed clinical net benefits across 18~80% threshold probabilities in the training cohort and 20~93% in the validation cohort. Clinically, early blastocyst formation was associated with superior embryo quality parameters (P<0.001), higher single embryo transfer rates (50.90% vs. 13.96%, P<0.001), and increased clinical (67.65% vs. 59.32%, P<0.001) and ongoing pregnancy rates (59.59% vs. 52.89%, P = 0.006), alongside a lower multiple pregnancy rate (22.50% vs. 31.93%, P<0.001). Multivariable regression confirmed early blastocyst formation as an independent predictor of pregnancy success (clinical pregnancy aOR=1.675, 95% CI: 1.287 ~ 2.180, P<0.001; ongoing pregnancy aOR=1.612, 95% CI: 1.249 ~ 2.080, P<0.001).

**Conclusion:**

The validated nomogram accurately predicts Day 4 early blastocyst formation using Day 3 embryo parameters. The presence of early blastocyst formation on Day 4 might indicate suitability for selective Day 4 embryo transfer.

## 1 Introduction

The determination of the optimal timing for embryo transfer has consistently remained a focal point of research in the field of assisted reproduction. Traditional day 3 cleavage-stage embryo transfer, with its shorter culture cycle and limited embryo exposure to the external environment, was formerly the mainstream choice (1). However, with the advancement of embryo culture technology and optimization of laboratory conditions, day 4 (morula, early blastocyst) and even day 5 (blastocyst) transfer have gradually become the trend (2–4). Many studies have shown that embryos at the blastocyst stage have completed important gene activation and cell differentiation, which confer stronger implantation ability and higher pregnancy rates (5, 6). Multiple clinical studies have reported that blastocyst transfer can improve implantation and pregnancy rates, and reduce the risk of multiple pregnancies (7, 8). However, it is noteworthy that some reports indicate a risk of embryo loss during blastocyst culture; especially in patients with limited embryo numbers, extended culture may reduce the number of transferable embryos and affect overall pregnancy outcomes (9). Therefore, clinical decisions should be individualized, with rational selection of transfer timing and embryo type according to patient conditions. Day 4 embryo transfer serves as a supplementary strategy, providing value for patients with low blastocyst formation rates or high risk of culture failure (10).

From a developmental biology perspective, day 4 embryos have completed the maternal-to-zygotic genome transition, and the activation of cell cycle checkpoints and apoptotic regulation mechanisms effectively reduces the incidence of aneuploidy and mosaicism (11). On day 4 after fertilization, uterine contractions decrease and endometrial receptivity increases. Lesny et al. (12) reported that uterine contraction waves gradually diminish two to four days post-oocyte retrieval, with only random waves observed on day 4. Moreover, prolonged *in vitro* culture may lead to abnormal expression of imprinted genes associated with apoptosis, oxidative stress, and gap junction formation (13). Compared to day 5 transfer, day 4 transfer shortens the culture period and reduces the risk of cycle cancellation due to extended culture. During compaction, enlargement of the perivitelline space enhances the safety of assisted hatching procedures (14). In natural conditions, fertilized embryos enter the uterine cavity around day 4, thus day 4 morula transfer better mimics physiological conditions (15). Clinical studies have shown that patients in the day 4 embryo transfer group have better age and embryo quality profiles than those in the day 5 transfer group, although clinical pregnancy and live birth rates are not statistically different between the two groups (16). Other studies have reported no significant differences in implantation rate, pregnancy rate, live birth rate, term delivery rate, or ectopic pregnancy rate between day 4 and day 5 transfers (3). Our previous research indicated that fresh day 4 embryo transfer achieves higher clinical pregnancy and live birth rates than day 5 transfer, with comparable maternal and neonatal outcomes, supporting the preferential use of day 4 transfer in clinical practice (17). Li et al. (11) found that the full-term birth rate following day 4 embryo transfer was significantly higher than that for day 5 transfer. Additional studies suggest that day 5 transfer may be associated with increased risks of preterm birth, congenital anomalies, monozygotic twins, and higher birth weight and gestational age, although the underlying mechanisms require further investigation (15). Furthermore, a higher proportion of live-born males has been reported following day 5 transfers, possibly due to embryo selection and the accelerated development of male embryos (18).

Day 4 embryo transfer, in which embryos have reached the morula or early blastocyst stage, combines the advantages of both day 3 and day 5 transfers. However, compaction on Day 4 is a dynamic process that may undergo oscillatory compaction and decompaction cycles, posing challenges for single-timepoint embryo assessment (19). In contrast, blastocoel cavity formation signifies successful passage through a critical developmental milestone and serves as a valid indicator of superior embryonic developmental potential. Our data reveal significantly higher clinical pregnancy and ongoing pregnancy rates compared to non-formation cases on Day 4 single embryo transfer. Predicting early blastocyst formation thus enables personalized transfer strategies. Patients with predicted high formation probability might benefit from selective Day 4 single embryo transfer, optimizing pregnancy rates while reducing multiple gestation risks.

## 2 Methods

### 2.1 Study design and participants

This single-center retrospective cohort study included patients who underwent *in vitro* fertilization and embryo transfer (IVF-ET) at Xingtai Meihe Reproductive and Genetic Hospital from January 2021 to December 2025. Inclusion criteria comprised: age 18~40 years, first or subsequent IVF-ET cycles, availability of transferable embryos with clearly defined transfer protocols, and complete clinical and laboratory records. Exclusion criteria included severe endocrine disorders affecting pregnancy outcomes, chromosomal abnormalities, severe uterine malformations, autoimmune diseases, or incomplete data. Ethical approval was obtained (Approval No. 2021-ER-09; initial approval: 2021-03-15; amendment for AI analysis: 2023-01-10), and all patients provided written informed consent in accordance with the Declaration of Helsinki.

### 2.2 Ovarian stimulation protocols

All patients underwent ovarian stimulation using standard gonadotropin-releasing hormone (GnRH) agonist or GnRH antagonist protocols. For those on the GnRH agonist protocol, a GnRH analogue (GnRHa, Leuprorelin, Shanghai Livzon Pharmaceutical Group) was administered subcutaneously at 2.50~3.75 mg from the mid-luteal phase. After successful down-regulation, recombinant follicle-stimulating hormone (FSH, Gensaiheng, Changchun GeneScience Pharmaceuticals) was injected subcutaneously at 150~225 IU to stimulate follicular growth.

For the GnRH antagonist protocol, stimulation commenced on menstrual cycle day 2 with recombinant FSH (Gensaiheng, Changchun GeneScience Pharmaceuticals) at 150~225 IU, with dosage adjusted according to follicular growth measured by transvaginal ultrasound. When the leading follicle reached 10~12 mm, daily GnRH antagonist (Cetrorelix, Hainan Hauron Pharmaceutical) was started at 0.25 mg. In the mid-to-late follicular phase, recombinant FSH (Gensaiheng, Changchun GeneScience Pharmaceuticals) was combined with human menopausal gonadotropin (hMG, Lepuode, Zhuhai Livzon Pharmaceutical Group) for further stimulation. When at least one third of follicles reached a diameter ≥18 mm, human chorionic gonadotropin (hCG, Zhuhai Livzon Pharmaceutical Group) was administered for triggering, at a dose of 5,000~10,000 IU, based on physician assessment. Oocyte retrieval was performed 36~37 hours post-hCG injection via transvaginal ultrasound-guided needle aspiration.

### 2.3 *In vitro* fertilization and embryo assessment

IVF/ICSI procedures were performed 3~4 hours after oocyte retrieval, with fertilization method determined by sperm parameters. For IVF, oocytes were cultured in 30~50 μL microdrops, each containing 1~2 oocytes and sperm at a concentration of 200,000/mL. Four to six hours post-fertilization, cumulus cells were removed. Embryos were cultured using a sequential culture system in EmbryoScope+ incubators (Vitrolife, A/S, Viby, Denmark) with EmbryoViewer software version 7.8.2 (Vitrolife, A/S, Denmark) installed.

Pronuclei number and scoring were assessed at (16 ± 18) hours post-fertilization using EmbryoViewer software. Cleavage-stage embryo morphology was evaluated at (68 ± 1) hours post-fertilization, and embryos were graded into three categories according to consensus: high-quality (≥7 cells, even size, ≤10% fragmentation), transferable (≥4 cells, even or moderate size, ≤25% fragmentation), and poor-quality (<4 cells and ≥25% fragmentation, or multinucleated and ≥50% fragmentation) (20). Additionally, artificial intelligence scoring was performed using the iDAScore V2.0 system (Vitrolife, Sweden) integrated with EmbryoScope+ timelapse incubators. This deep learning algorithm analyzed 113 morphokinetic parameters (e.g., time to syngamy, blastomere symmetry patterns) through a 3D convolutional neural network, generating scores from 1.0 (lowest developmental potential) to 9.0 (highest). All images were acquired at 10-minute intervals under stable culture conditions (37 °C, 6% CO_2_, 5% O_2_).

On the fourth day (92 ± 2 hours post-fertilization), the early blastocyst exhibited a visible blastocoel cavity occupying no more than 50% of the embryo’s volume, with distinguishable inner cell mass and trophectoderm precursors (20). The compaction status was classified as fully compacted (complete cell fusion), partially compacted (50~90% fusion), or non-compacted (<50% fusion). Two senior embryologists conducted the assessments, with any discrepancies resolved by a third evaluator, who was blinded to the results.

In this study, Day 4 embryo transfer was performed unless the number of transferable embryos on Day 3 did not exceed the planned number for transfer, in which case one or two embryos were selected for fresh transfer on Day 4. When ≥4 high-quality embryos were available on Day 3, extended culture to Day 5 was prioritized for blastocyst transfer.

### 2.4 Grouping and outcome definitions

Participants were randomly allocated to training (n=1,789) and validation (n=768) sets at a 7:3 ratio using computer-generated randomization with stratification by female age (± 2 years) and infertility type (primary/secondary) to ensure balanced group distribution.

The primary outcome was the early blastocyst formation rate. Secondary outcomes included: clinical pregnancy rate (number of cycles with intrauterine gestational sac confirmed by transvaginal ultrasound showing yolk sac or cardiac activity per embryo transfer cycle ×100%), ongoing pregnancy rate (number of pregnancies progressing beyond 12 weeks with viable fetus on ultrasound per embryo transfer cycle ×100%), and early miscarriage rate (number of pregnancy losses ≤12 weeks including biochemical pregnancy, blighted ovum, and spontaneous abortion per clinical pregnancy ×100%). Ultrasound assessments were independently performed by two senior physicians, with discrepancies resolved by a third blinded evaluator, excluding ectopic pregnancies and iatrogenic terminations.

### 2.5 Statistical analysis

Categorical variables were analyzed using the chi-square test or Fisher’s exact test. The normality of continuous variables was tested using the Shapiro-Wilk test; normally distributed data were expressed as mean ± standard deviation and compared using the t-test or ANOVA, while non-normally distributed data were expressed as median (interquartile range) and compared using the Mann-Whitney U test.

A tiered validation approach was used in the statistical analysis to ensure robustness. Stratified random sampling (SPSS 26.0) was used for dataset partitioning, allocating 70% of cases to the training set and 30% to the test set, while ensuring identical distributions of key prognostic factors (female age, AMH level, and infertility duration) between the sets. Receiver operating characteristic (ROC) curve analysis was employed to evaluate predictive ability, with the optimal cut-off determined by the Youden index.

For early blastocyst formation on day 4, univariate analysis was conducted to identify significant variables, which were then included in binary logistic regression to calculate odds ratios (OR) and 95% confidence intervals (CI) for independent predictors. A nomogram prediction model for early blastocyst formation was constructed based on independent predictors. The predictive accuracy of the model was evaluated using receiver operating characteristic (ROC) curve analysis, and the area under the curve (AUC) was calculated to assess the model’s discriminative ability. The calibration of the model was assessed using the Hosmer-Lemeshow goodness-of-fit test. Internal validation was performed using bootstrapping with 1000 resamples to assess the stability and reliability of the model estimates, while external validation was performed using an independent validation dataset to evaluate the model’s generalizability. It should be noted that this does not constitute traditional external validation, which typically involves testing on data from a different site. All statistical analyses were performed using SPSS 26.0 and MedCalc software, with P < 0.05 considered statistically significant.

## 3 Results

A total of 2,557 patients scheduled for fresh Day 4 embryo transfer were enrolled according to predefined inclusion/exclusion criteria and randomly allocated to training (n=1,789) and validation (n=768) sets at a 7:3 ratio. Early blastocyst formation occurred in 803 patients (44.89%) versus 986 non-formation cases (55.11%) in the training set, and 339 (44.14%) versus 429 (55.86%) in the validation set. Baseline characteristics, embryonic outcomes, and clinical outcomes showed no significant differences between training and validation sets (**Supplementary Table 1**).

### 3.1 Comparative analysis of baseline characteristics between the early blastocyst formation and non-formation groups within the training set

The formation group exhibited younger female age (median 31 vs. 32 years, P = 0.001), superior ovarian reserve (AMH: 3.52 vs. 3.13 ng/mL; AFC: 17 vs. 16, both P<0.001), higher basal LH (4.53 vs. 4.14 IU/L, P = 0.007), and greater PCOS prevalence (15.69% vs. 11.05%, P = 0.004). Male partners were younger (31 vs. 32 years, P = 0.004) with better semen parameters: higher sperm concentration (53.63 vs. 48.45×10^6^/mL, P = 0.009), progressive motility (44.12% vs. 42.00%, P<0.001), and lower DFI (11.28% vs. 12.22%, P = 0.014). Treatment response favored the formation group, evidenced by lower gonadotropin requirements (P = 0.004), higher hCG-day estradiol (2751 vs. 2528 pg/mL, P<0.001), and reduced ICSI usage (16.81% vs. 28.89%, P<0.001). No differences existed in infertility duration, BMI (female/male), primary infertility rate, basal FSH, sperm normal morphology, stimulation days, or hCG-day progesterone (P>0.05) (**Table 1**).

**Table 1:** Comparison of baseline characteristics between patients with and without early blastocyst formation on day 4.

Variables	Without blastocyst (n = 986)	With blastocyst (n = 803)	Statistic	P-value
Female age(y)	32.00 (28.00, 35.00)	31.00 (28.00, 34.00)	Z=-3.29	0.001
Duration of infertility(y)	4.00 (2.00, 6.00)	3.00 (2.00, 6.00)	Z=-0.88	0.378
Female BMI (kg/m2)	24.20 (21.80, 26.90)	24.00 (21.60, 26.90)	Z=-0.32	0.750
Primary infertility rate (%)	422 (42.80)	313 (38.98)	χ²=2.67	0.102
PCOS (%)	109 (11.05)	126 (15.69)	χ²=8.34	0.004
Basic FSH(IU/L)	6.54 (5.33, 7.85)	6.44 (5.44, 7.75)	Z=-0.53	0.593
Basic LH(IU/L)	4.14 (3.03, 6.10)	4.53 (3.16, 6.71)	Z=-2.68	0.007
AMH(ng/ml)	3.13 (1.99, 4.93)	3.52 (2.28, 5.40)	Z=-3.65	<0.001
AFC	16.00 (12.00, 20.00)	17.00 (13.00, 23.00)	Z=-3.59	<0.001
Male age(y)	32.00 (29.00, 35.00)	31.00 (28.00, 34.00)	Z=-2.84	0.004
Male BMI (kg/m2)	25.35 (22.62, 28.09)	25.61 (22.98, 28.19)	Z=-0.94	0.346
Sperm concentration (10^6/mL)	48.45 (25.44, 80.50)	53.63 (31.45, 83.05)	Z=-2.60	0.009
Progressive motility (%)	42.00 (29.26, 53.28)	44.12 (34.30, 55.67)	Z=-3.34	<0.001
Sperm with normal morphology (%)	3.50 (1.97, 5.00)	3.67 (2.00, 5.00)	Z=-1.12	0.262
DFI (%)	12.22 (8.00, 18.20)	11.28 (7.27, 17.52)	Z=-2.45	0.014
Ovarian stimulation protocol			χ²=0.23	0.630
GnRH agonist	905 (91.78)	742 (92.40)		
GnRH antagonist	81 (8.22)	61 (7.60)		
Total Gn dose (IU)	2700.00 (2206.25, 3300.00)	2587.50 (2075.00, 3181.25)	Z=-2.84	0.004
Duration of Gn stimulation(day)	11.00 (10.00, 12.00)	11.00 (10.00, 12.00)	Z=-1.47	0.141
Estradiol, hCG day(ng/ml)	2528.00 (1722.50, 3509.25)	2751.00 (1882.00, 3814.50)	Z=-3.35	<0.001
Progestin, hCG day(ng/ml)	0.71 (0.52, 0.91)	0.70 (0.53, 0.89)	Z=-0.42	0.674
ICSI rate (%)	284 (28.89)	135 (16.81)	χ²=35.91	<0.001

Data are M (Q_1_, Q_3_) or n.

BMI, body mass index. PCOS, polycystic ovary syndrome. FSH, follicle-stimulating hormone. LH, luteinizing hormone. AMH, anti-mullerian hormone. AFC, antral follicle count. DFI, DNA fragmentation index. ICSI, intracytoplasmic sperm injection. Gn, Gonadotropin. GnRH, gonadotropin-releasing hormone. hCG, Human Chorionic Gonadotropin.

### 3.2 Predictive value of day 3 embryo parameters for early blastocyst formation on day 4

**Figure 1:**
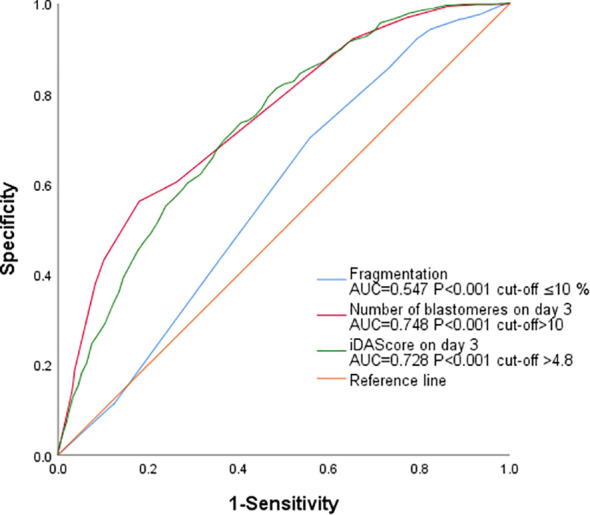
Receiver operating characteristic (ROC) analysis of day 3 embryo parameters predicting early blastocyst formation on day 4, with optimal cut-offs determined by Youden index optimization.

After controlling for variables including female age, PCOS, basal LH, AMH, AFC, male age, sperm concentration, DFI, total gonadotropin dose, estradiol level, and ICSI rate, the number of blastomeres on Day 3 (OR = 1.456, 95% CI: 1.375~1.541, P<0.001) and the iDAScore (OR = 1.428, 95% CI: 1.318~1.548, P<0.001) were identified as independent and significant predictors for early blastocyst formation on Day 4. Fragmentation demonstrated minimal clinical relevance despite its statistical significance (OR = 1.020, 95% CI: 1.001~1.038, P = 0.039). Parameters exhibiting non-significance (all P>0.05): Zygote Score (Z-score): Groups Z2, Z3, and Z4 exhibited no predictive advantage over Z1 (P>0.05). The “Fair” and “Poor” grades did not significantly differ from the “Good” grade. Blastomere Symmetry: “Moderately uneven” and “Severely uneven” symmetry exhibited no predictive value (P>0.05) (**Table 2**). ROC analysis indicated that the number of blastomeres, iDAScore, and fragmentation on Day 3 predicted early blastocyst formation on Day 4 with AUC values of 0.748, 0.728, and 0.547 (P<0.001), respectively and the optimal cut-off values determined by the Youden index were >10, >4.8, and ≤10% (**Figure 1**).

**Table 2:** Binary logistic regression analysis was performed to evaluate the association between day 3 embryo parameters and early blastocyst formation on day 4.

Variables	Coefficient	Standard error	p-value	Odds ratios(95% CI)
Z-score				
Z2 vs.Z1	0.439	0.639	0.492	1.551(0.443~5.432)
Z3 vs.Z1	0.179	0.637	0.779	1.196(0.343~4.171)
Z4 vs.Z1	0.199	0.673	0.768	1.220(0.326~4.566)
Number of blastomeres	0.375	0.029	< 0.001	1.456(1.375~1.541)
Morphological grades for nucleolar precursor bodies				
Fair vs.Good	0.107	0.184	0.562	1.113(0.776~1.597)
Bad vs.Good	-0.028	0.408	0.946	0.973(0.437~2.164)
Fragmentation	0.019	0.009	0.039	1.020(1.001~1.038)
Blastomere symmetry				
Moderate vs. Even	0.107	0.184	0.562	1.113(0.776~1.597)
Poor vs. Even	-0.028	0.408	0.946	0.973(0.437~2.164)
iDAScore	0.356	0.041	<0.001	1.428(1.318~1.548)

Adjusted for female age, PCOS, basal LH, AMH, AFC, male age, sperm concentration, DFI, total gonadotropin dose, estradiol level, and ICSI status. These covariates demonstrated statistically significant differences in baseline characteristics as detailed in **Table 1**.

### 3.3 Clinical characteristics associated with early blastocyst formation on day 4

Patients with early blastocyst formation on Day 4 demonstrated superior laboratory and clinical outcomes compared to non-formation cases (**Table 3**). The formation group yielded significantly more oocytes (median: 13.00 vs. 12.00, P<0.001) and normal fertilized zygotes (8.00 vs. 7.00, P<0.001). Their embryos exhibited markedly better quality on Day 3, with a greater number of transferable embryos (2.00 vs. 1.00, P<0.001), high-quality embryos (7.00 vs. 5.00, P<0.001), embryos with >10 blastomeres (1.00 vs. 0.00, P<0.001), and embryos with fragmentation ≤10% (5.00 vs. 4.00, P<0.001). Notably, they also possessed a significantly higher number of embryos with an iDAScore >4.8 (4.00 vs. 2.00, P<0.001). Endometrial thickness was comparable between the groups (11.20 mm vs. 11.00 mm, P = 0.262). Clinically, the formation group had a dramatically higher rate of single embryo transfer (50.90% vs. 13.96%, P<0.001), which was associated with superior clinical pregnancy (67.65% vs. 59.32%, P<0.001) and ongoing pregnancy rates (59.59% vs. 52.89%, P = 0.006), alongside a lower multiple pregnancy rate (22.50% vs. 31.93%, P<0.001). The early miscarriage rate did not differ significantly between the groups (11.91% vs. 10.85%, P = 0.583).

**Table 3:** Comparison of embryo outcomes between patients with and without early blastocyst formation on day 4.

Variables	Without blastocyst (n = 986)	With blastocyst (n = 803)	Statistic	P-value
Number of oocytes retrieved(n)	12.00 (8.00, 15.00)	13.00 (9.00, 17.00)	Z=-4.62	<0.001
Number of normal fertilization(n)	7.00 (5.00, 10.00)	8.00 (6.00, 11.00)	Z=-6.69	<0.001
Number of transferable embryos on the third day(n)	1.00 (0.00, 3.00)	2.00 (1.00, 3.00)	Z=-3.52	<0.001
Number of high-quality embryos on the third day(n)	5.00 (4.00, 7.00)	7.00 (5.00, 9.00)	Z=-10.09	<0.001
Number of embryos with >10 blastomeres, M (Q_1_, Q_3_)	0.00 (0.00, 1.00)	1.00 (0.00, 2.00)	Z=-14.57	<0.001
Number of embryos with fragmentation ≤ 10%, M (Q_1_, Q_3_)	4.00 (2.00, 6.00)	5.00 (3.00, 7.00)	Z=-7.01	<0.001
Number of embryos with iDAScore>4.8(n)	2.00 (1.00, 4.00)	4.00 (2.00, 5.00)	Z=-7.63	<0.001
Endometrial thickness, M (Q_1_, Q_3_)	11.00 (9.60, 12.70)	11.20 (10.00, 13.00)	Z=-1.12	0.262
Number of embryos transferred			χ²=269.40	<.001
Single (%)	128 (13.96)	398 (50.90)		
Double (%)	789 (86.04)	384 (49.10)		
Clinical pregnancy rate (%)	544 (59.32)	529 (67.65)	χ²=12.57	<.001
Multiple Pregnancy Rate (%)	175 (31.93)	119 (22.50)	χ²=12.08	<.001
Ongoing pregnancy rate (%)	485 (52.89)	466 (59.59)	χ²=7.69	0.006
Early miscarriage rate (%)	59 (10.85)	63 (11.91)	χ²=0.30	0.583

Binary logistic regression analysis, adjusted for 15 covariates including female age, ovarian reserve markers (AMH, AFC), PCOS status, sperm parameters (concentration, PR, DFI), and treatment characteristics (Gn protocol, total Gn dose, ICSI usage), revealed that early blastocyst formation on Day 4 was a significant independent predictor for superior pregnancy outcomes. Specifically, the presence of an early blastocyst was associated with a 1.675 fold increased odds of achieving a clinical pregnancy (Adjusted OR = 1.675, 95% CI: 1.287–2.180, P < 0.001) and a 1.612 fold increased odds of achieving an ongoing pregnancy (Adjusted OR = 1.612, 95% CI: 1.249–2.080, P < 0.0001) when compared to cycles with only fully compacted embryos (**Table 4**).

**Table 4:** The impact of early blastocyst formation relative to complete fusion embryos on pregnancy outcomes was assessed using binary logistic regression analysis.

Variables	Clinical pregnancy rate		Ongoing pregnancy rate	
	Adjust odds ratios(95%CI)	P-value	Adjust odds ratios (95%CI)	P-value
Female age	0.985(0.945~1.027)	0.491	0.972(0.934~1.010)	0.178
PCOS	1.51(0.960~2.375)	0.074	1.895(1.219~2.950)	0.005
Basic LH	1.004(0.988~1.021)	0.598	1.009(0.992~1.030)	0.294
AMH	1.007(0.941~1.077)	0.848	0.98(0.919~1.050)	0.547
AFC	0.994(0.966~1.021)	0.648	0.998(0.971~1.030)	0.881
Male age	1.006(0.967~1.046)	0.761	1.016(0.978~1.060)	0.416
Sperm concentration	0.998(0.996~1.001)	0.245	0.998(0.995~1.000)	0.102
PR	1(0.992~1.008)	0.984	0.997(0.989~1.000)	0.431
DFI	1.009(0.996~1.022)	0.188	1.006(0.993~1.020)	0.361
GnRH antagonist protocol	0.928(0.568~1.516)	0.765	0.873(0.543~1.400)	0.574
Total Gn dose	1.000(1.000~1.000)	0.565	1.000(1.000~1.000)	0.736
Estradiol	1.000(1.000~1.000)	0.962	1.000(1.000~1.000)	0.903
Progestin	0.597(0.376~0.949)	0.029	0.687(0.443~1.070)	0.094
Number of oocytes retrieved	0.991(0.962~1.021)	0.543	0.988(0.96~1.020)	0.423
ICSI	0.881(0.632~1.229)	0.457	0.813(0.587~1.130)	0.211
Number of embryos transferred:	1.529(1.148~2.037)	0.004	1.558(1.177~2.060)	0.002
With blastocyst on Day 4	1.675(1.287~2.180)	< 0.001	1.612(1.249~2.080)	< 0.001

CI, Confidence Interval; AMH, anti-mullerian hormone; AFC, Antral Follicle Count; PCOS, polycystic ovary syndrome; DFI, DNA fragmentation index; ICSI, intracytoplasmic sperm injection; Gn, Gonadotropin; hCG, Human Chorionic Gonadotropin.

Comparison of outcomes for single good-quality embryo transfers at different developmental stages revealed no significant differences in endometrial thickness among groups (P>0.05). Clinical pregnancy rates were significantly higher for Day 4 single morula transfers (55.95%), Day 4 single early blastocyst transfers (64.19%), and Day 5 single blastocyst transfers (52.51%) compared to Day 3 single cleavage-stage embryo transfers (35.29%; P< 0.05). Notably, Day 4 single early blastocyst transfers achieved the highest clinical pregnancy rate (64.19%), significantly exceeding all other groups. For ongoing pregnancy rates, Day 4 single early blastocyst transfers (54.85%) were significantly superior to both Day 3 (29.41%) and Day 5 transfers (44.60%; P< 0.05). However, no significant differences existed between Day 4 morula and Day 5 blastocyst transfers in clinical or ongoing pregnancy rates (P> 0.05). (Detailed data in **Supplementary Table 2**).

### 3.4 Binary logistic regression analysis of factors influencing early blastocyst formation on day

**Table 5** presents the results of a multivariate binary logistic regression analysis identifying independent predictors of early blastocyst formation on day 4. After adjusting for multiple clinical, laboratory, and embryological variables, only two parameters retained significant independent predictive value: the number of embryos with >10 blastomeres on day 3 (Adjusted OR = 1.668, 95% CI: 1.374~2.025, p<0.001) and the number of embryos with an iDAScore >4.8 on day 3 (Adjusted OR = 1.369, 95% CI: 1.190~1.575, p<0.001). In contrast, other factors including female age, AMH, AFC, PCOS status, male age, sperm DNA fragmentation index (DFI, p=0.051), progressive sperm motility, total gonadotropin dose, estradiol level on hCG day, ICSI utilization, number of oocytes retrieved, and conventional day 3 embryo parameters (total transferable embryos, number of high-quality embryos, and number of embryos with fragmentation ≤10%) were not statistically significant independent predictors in this comprehensive model (p>0.05 for all).

**Table 5:** Binary logistic regression analysis of factors influencing early blastocyst formation on day 4.

Variables	Coefficient	Standard error	P-value	Adjust odds ratios (95% CI)
Female age	-0.016	0.04	0.690	0.984 (0.910 ~ 1.065)
AMH	0.016	0.076	0.839	1.016 (0.875 ~ 1.179)
AFC	-0.017	0.026	0.526	0.983 (0.934 ~ 1.035)
PCOS	0.109	0.409	0.789	1.115 (0.501 ~ 2.484)
Male age	-0.032	0.039	0.400	0.968 (0.898 ~ 1.044)
DFI	-0.023	0.012	0.051	0.977 (0.955 ~ 1.000)
Progressive motility	-0.009	0.008	0.246	0.991 (0.975 ~ 1.007)
Total Gn dose	<0.001	<0.001	0.478	1.000 (1.000 ~ 1.000)
Estradiol	<0.001	<0.001	0.065	1.000 (1.000 ~ 1.000)
ICSI	-0.111	0.333	0.738	0.895 (0.466 ~ 1.718)
Number of oocytes retrieved	-0.025	0.033	0.446	0.976 (0.915 ~ 1.040)
Number of transferable embryos, day 3	-0.051	0.086	0.554	0.950 (0.803 ~ 1.125)
Number of high-quality embryos, day 3	0.017	0.07	0.812	1.017 (0.886 ~ 1.167)
Number of embryos with >10 blastomeres	0.512	0.099	<0.001	1.668 (1.374 ~ 2.025)
Number of embryos with fragmentation ≤ 10%	0.03	0.073	0.678	1.031 (0.893 ~ 1.190)
Number of embryos with iDAScore>4.8	0.314	0.072	<0.001	1.369 (1.190 ~ 1.575)

CI, Confidence Interval; AMH, anti-mullerian hormone; AFC, Antral Follicle Count; PCOS, polycystic ovary syndrome; DFI, DNA fragmentation index; ICSI, ntracytoplasmic sperm injection; Gn, Gonadotropin; hCG, Human Chorionic Gonadotropin.

### 3.5 Predictive model development and validation for day 4 early blastocyst formation

**Figure 2:**
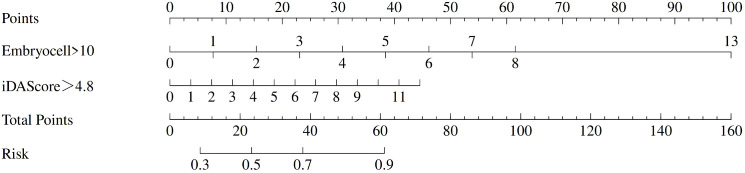
Nomogram prediction model for early blastocyst formation on day 4.

Multivariable binary logistic regression analysis identified two independent predictors of early blastocyst formation on Day 4: the number of embryos with >10 blastomeres on Day 3 and the number of embryos with iDAScore >4.8 on Day 3. Based on these predictors, a nomogram model was developed to quantitatively estimate the probability of early blastocyst formation. The model integrated two key variables: embryos with >10 blastomeres on Day 3 and the number of embryos with iDAScore >4.8. Each variable was assigned a points score, and the total points corresponded to a predicted probability of early blastocyst formation, providing a practical tool for clinical decision-making (**Figure 2**).

**Figure 3:**
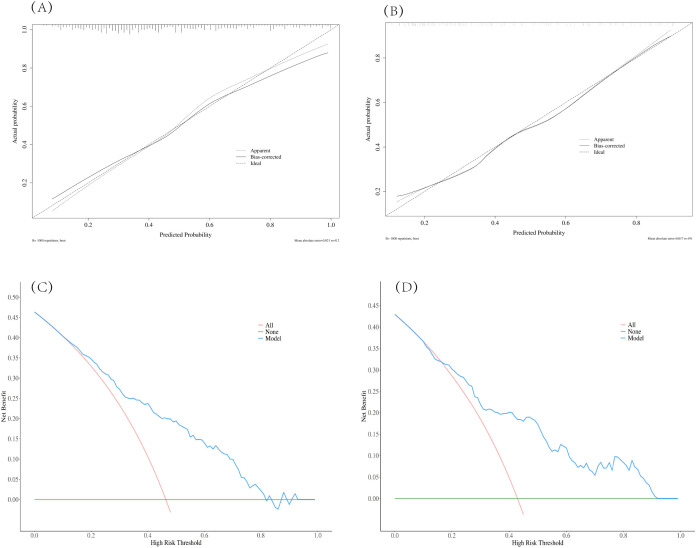
Assessment of nomogram performance: calibration and decision curve analysis. Calibration curves and decision curve analysis (DCA) were used to evaluate the nomogram’s predictive accuracy and clinical utility in the training and validation cohorts. The calibration curves demonstrate good agreement between the predicted probability and the actual incidence of transferable blastocyst formation in both the training **(A)** and validation **(B)** cohorts. DCA indicates that the use of the nomogram provides a clinical net benefit across threshold probabilities of 18–80% in the training cohort **(C)** and 20–93% in the validation cohort **(D)**.

The nomogram demonstrated robust performance, with an AUC of 0.782 (95% CI: 0.738~0.826) in the training set and 0.773 (95% CI: 0.705~0.841) in the validation set, indicating consistent discriminative ability (**Table 6**). Calibration curves revealed excellent agreement between predicted probabilities and observed outcomes in both cohorts. Decision curve analysis confirmed the clinical utility of the model, showing net benefits across threshold probabilities of 18~80% (training) and 20~93% (validation), supporting its applicability in clinical settings where probability thresholds between 20% and 90% are relevant for decision-making (**Figure 3**).

The model achieved balanced performance metrics, with sensitivity of 65.4~65.9%, specificity of 79.6~81.7%, and accuracy of 73.1~74.9% across datasets. These results validate the nomogram as a reliable tool for predicting Day 4 early blastocyst formation, leveraging objectively measurable Day 3 embryo parameters to guide embryo selection strategies in clinical practice (**Table 6**).

**Table 6:** Performance metrics of the predictive model for early blastocyst formation on day 4.

Data	AUC (95%CI)	Accuracy (95%CI)	Sensitivity (95%CI)	Specificity (95%CI)	PPV (95%CI)	NPV (95%CI)
Train	0.782 (0.738-0.826)	0.731 (0.685-0.773)	0.654 (0.587 - 0.722)	0.796 (0.743 - 0.849)	0.735 (0.669 - 0.802)	0.727 (0.671 - 0.783)
Test	0.773 (0.705-0.841)	0.749 (0.681-0.809)	0.659 (0.556 - 0.761)	0.817 (0.744 - 0.889)	0.730 (0.629 - 0.831)	0.761 (0.683 - 0.838)

## 4 Discussion

This study confirms that the predictive model for Day 4 early blastocyst formation, constructed based on Day 3 blastomere number and iDAScore, exhibits high discriminative accuracy and clinical applicability. After adjusting for confounders, the presence of early blastocysts on Day 4 significantly increased the proportion of single embryo transfers (50.9% vs. 15.3%, P<0.001) and improved clinical pregnancy rates (67.8% vs. 61.2%, P = 0.006) and ongoing pregnancy rates​ (64.9% vs. 58.6%, P = 0.011). These findings support the use of this model to guide selective Day 4 single embryo transfer in clinical practice.

Day 4 embryo transfer serves as a flexible strategy that may yield outcomes comparable to Day 5 blastocyst transfer (21). However, compaction on Day 4 is a dynamic process that may undergo oscillatory compaction and decompaction cycles, posing challenges for single-timepoint embryo assessment (19). Furthermore, distinguishing exclusionary, extrusion, and mixed compaction patterns necessitates time-lapse imaging for dynamic monitoring (19, 22). This study compared clinical outcomes between cycles with and without Day 4 early blastocyst formation. Key findings demonstrate that extending culture to blastocyst stages enhances selection of high-potential embryos, optimizes transfer candidates, and improves pregnancy rates. Notably, transferring Day 4 early blastocysts achieved significantly higher clinical pregnancy (67.77% vs. 61.19%, P = 0.006) and ongoing pregnancy rates (64.95% vs. 58.58%, P = 0.011) than high-quality Day 5 blastocyst transfers. Single Day 4 early blastocyst transfer emerges as a superior strategy to Day 4 morula transfer, reducing multiple gestation risk (22.50% vs. 31.93%, P<0.001) without compromising pregnancy success (**Supplementary Table 1**). Utilizing early blastocyst formation as a biomarker simplifies Day 4 embryo selection while maintaining high clinical efficacy. Consequently, developing predictive models for Day 4 blastulation is clinically imperative to enable personalized transfer strategies.

To determine under what circumstances selective day 4 transfer could be ensured by early blastocyst formation, we first compared day 3 embryo parameters between the day 4 early blastocyst group and the non-early blastocyst group. Previous research has shown that analyzing cell number and fragmentation in relation to blastocyst formation rate can help improve live birth rates in cleavage-stage embryo transfer (23). Recent studies have established nomogram prediction models for blastocyst formation based on day 3 blastomere number, uniformity, fragmentation, and morphology score, achieving an AUC of 0.742 (24). However, previous studies have focused on day 5 and day 6 blastocyst formation factors, with little literature on day 4 early blastocyst formation. Similarly, we found significant differences in day 3 morphological score, blastomere number, fragmentation, and uniformity between the early blastocyst group and the non-early blastocyst group. Morphological scoring is the main traditional method for embryo selection, but it is subject to considerable subjectivity and inconsistent standards. Time-lapse imaging has brought breakthroughs in embryo observation, recording the full course of embryo development *in vitro*; its derived morphokinetic parameters have also contributed to embryo selection, and some studies have reported their close relation to embryo euploidy (25). Some research teams have developed the KID3 scoring system based on pronuclear fading time, 2-cell, 3-cell, 5-cell, and 8-cell timings, showing significant differences in blastocyst formation rate, high-quality blastocyst rate, and pregnancy rate for embryos with different scores (26). However, marking morphokinetic parameters is labor-intensive. The introduction of AI scoring has brought a breakthrough to embryo evaluation. Studies comparing AI models and manually annotated morphokinetic systems for predicting live birth found that manual annotation could better predict outcomes, but combining with AI models further improved accuracy (27). Although AI predictive power still needs improvement, AI scores have been shown to be significantly associated with live birth probability in single embryo transfer cycles. Satoshi Ueno et al. reported that a deep learning-based annotation-free embryo scoring system could effectively reduce miscarriage rates and improve live birth rates (28). Embryos with low AI scores that are aneuploid or mosaic are associated with lower live birth rates, indicating the potential of annotation-free AI systems as decision support tools to help select embryos with poor PGT-A results (29). There is a statistically significant association between AI score and embryo euploidy, showing great potential for noninvasive euploidy screening (30). Therefore, AI not only brings convenience but also serves as a practical tool to improve embryo selection accuracy. The Vitrolife iDAScore V1 system provides fully automated annotation-free scoring for blastocysts, mainly based on implantation ability, scoring from 1 to 9.9 (31). Studies have confirmed the significant association of iDAScore V1 with blastocyst euploidy rate (30, 32), and retrospective studies also confirm its relevance to live birth after single blastocyst thaw transfer (28, 33). The recently developed iDAScore V2 system scores cleavage-stage embryos using a 3D convolutional neural network, based on implantation ability (34, 35). Studies have shown that iDAScore V2 provides good guidance for single cleavage-stage embryo transfer (36). However, the integration of AI scoring with traditional morphology and its predictive value for blastocyst outcomes still require further research and validation. In this study, we used the iDAScore V2 system for embryo quantitative assessment, and found that AI score and day 3 cell number were independent predictors of day 4 early blastocyst formation. ROC analysis showed that AI score predicted day 4 early blastocyst formation with an AUC of 0.728 (P<0.001), and the optimal cut-off value determined by the Youden index was > 4.8. Embryos with iDAScore >4.8 were defined as high AI score embryos. Traditionally, an embryo cell count of 8 on Day 3 was considered optimal (20). However, recent studies on Day 4 embryo selection indicate that 10–12 cells on Day 3​ yield the best outcomes (37). For thawed blastocysts, embryos with 9–13 cells on Day 3​ outperform those with <7 cells, with higher cell counts correlating with increased pregnancy rates (38). On Day 4 post-fertilization, embryos typically reach the morula stage. Early blastocyst formation represents accelerated development, supported by embryokinetic studies confirming a significant correlation between blastocoel cavity formation timing and the 8-cell stage transition. This explains why >10 cells on Day 3​ emerged as an independent predictor of Day 4 early blastocyst formation in our study (adjusted OR = 1.668, 95% CI: 1.374–2.025, P<0.001).

Next, we compared clinical data between groups with and without day 4 early blastocyst formation. Studies have shown that the number of mature oocytes and ICSI fertilization are important factors for blastocyst formation rate in selective day 5 transfer cycles (39). Other studies report that female age, gonadotropin dosage, and fertilization method are associated with blastocyst quality (40, 41). Young women who received appropriate gonadotropin dosages and IVF fertilization are suitable for blastocyst transfer (42). However, there is little literature on day 4 early blastocyst formation factors. Our results showed that the group with blastocyst formation had lower female age, primary infertility, total Gn dosage and Gn days than the group without blastocyst formation; while AMH, trigger day E2, number of oocytes retrieved, normal fertilization number, transferable embryos, high-quality embryos, and high AI score embryos were all significantly increased. Interestingly, male age was lower and semen density and forward motile sperm were higher in the early blastocyst group than the non-early blastocyst group. After adjusting for confounders, further binary logistic regression analysis found that Number of embryos with >10 blastomeres and number of high AI score embryos were independent predictors of day 4 early blastocyst formation. A nomogram prediction model for Day 4 early blastocyst formation was constructed using these independent predictors. This model was validated to present good discrimination and calibration, and obtained clinical net benefits both in the training and validation cohorts.

This single-center retrospective cohort study is subject to selection and information biases inherent to its design. The extended recruitment period (2018–2025) may have resulted in incomplete records for some participants. Crucially, both Day 4 and Day 5 transfers were elective procedures, leading to significant baseline differences between groups. Although we conducted subgroup analyses and multivariable regression to mitigate confounding, residual bias cannot be excluded. While this study demonstrates that early blastocyst formation identifies optimal candidates for Day 4 transfer, our predictive model relies on iDAScore assessments requiring time-lapse imaging systems. Centers without such technology should develop institution-specific models aligned with their culture conditions and assessment protocols. Additionally, the exclusion of genetic screening (PGT-A), metabolomic profiling, and other multi-dimensional data limits comprehensive evaluation of embryonic developmental potential. Future multi-center prospective trials integrating these parameters are warranted to validate and refine embryo selection strategies.

In conclusion, the validated nomogram effectively predicts early blastocyst formation on Day 4 using Day 3 embryo parameters, specifically blastomere number greater than 10 and iDAScore exceeding 4.8, with notable discriminative accuracy. The presence of early blastocyst formation on Day 4 may correlate with suitability for selective embryo transfer on the same day, as reflected by significantly higher ongoing pregnancy rates. This model is expected to optimize embryo selection and single-embryo transfer decision-making by prospectively identifying embryos that are suitable for Day 4 transfer, while embryos predicted with lower potential may be prioritized for Day 3 transfer or Day 5 transfer.

## Data Availability

The data analyzed in this study is subject to the following licenses/restrictions: The datasets generated and analyzed during the current study are not publicly available, since the dataset will be used for other retrospective analyses. The data are available from the corresponding author upon reasonable request. Requests to access these datasets should be directed to Yasong, Geng. mhsz@mhrepro.cn.

## Supplementary material

Supplementary Table 1 Comparison of baseline characteristics between training and validation cohorts. Data are M (Q_1_, Q_3_) or n. BMI, body mass index. PCOS, polycystic ovary syndrome. FSH, follicle-stimulating hormone. LH, luteinizing hormone. AMH, anti-mullerian hormone. AFC, antral follicle count. DFI, DNA fragmentation index. ICSI, intracytoplasmic sperm injection. Gn, Gonadotropin. hCG, Human Chorionic Gonadotropin.

Supplementary Table 2 Comparison of pregnancy outcomes following single high-quality embryo transfer on different days. Data are M (Q_1_, Q_3_) or n. ^a^Statistically significant compared with cleavage on day 3; ^b^Statistically significant compared with full compaction on day4; ^c^Statistically significant compared with early blastocyst on day4.
