# Supplementary material for: Development and validation of a predictive model for early blastocyst formation on day 4 post-fertilization
Source: Front Endocrinol (Lausanne). 2026 Apr 1;17:1752963. doi: 10.3389/fendo.2026.1752963 (PMC13079057; doi:10.3389/fendo.2026.1752963)
Supplement: Supplementary Table 1 — Comparison of baseline characteristics between training and validation cohorts. Data are M (Q1, Q3) or n. BMI, body mass index. PCOS, polycystic ovary syndrome. FSH, follicle-stimulating hormone. LH, luteinizing hormone. AMH, anti-mullerian hormone. AFC, antral follicle count. DFI, DNA fragmentation index. ICSI, intracytoplasmic sperm injection. Gn, Gonadotropin. hCG, Human Chorionic Gonadotropin. [file Table1.docx]

| **Supplementary Table S1.** Comparison of Baseline Characteristics Between Training and Validation Cohorts | | | | |
| --- | --- | --- | --- | --- |
| **Variables** | **test (n = 768)** | **train (n = 1789)** | **Statistic** | **P** |
| Female age(y) | 31.00 (28.00, 34.00) | 31.00 (28.00, 34.00) | Z=-0.15 | 0.881 |
| Duration of infertility(y) | 3.00 (2.00, 6.00) | 4.00 (2.00, 6.00) | Z=-0.30 | 0.763 |
| Female BMI (kg/m2) | 24.20 (21.80, 27.20) | 24.10 (21.70, 26.90) | Z=-0.96 | 0.338 |
| Primary infertility rate (%) | 309 (40.23) | 735 (41.08) | χ²=0.16 | 0.689 |
| PCOS (%) | 112 (14.58) | 235 (13.14) | χ²=0.96 | 0.327 |
| Basic FSH(IU/L) | 6.54 (5.38, 8.00) | 6.50 (5.37, 7.80) | Z=-0.64 | 0.525 |
| Basic LH(IU/L) | 4.41 (2.96, 6.67) | 4.30 (3.08, 6.41) | Z=-0.23 | 0.816 |
| AMH (ng/ml) | 3.36 (2.21, 4.89) | 3.29 (2.10, 5.07) | Z=-0.28 | 0.776 |
| AFC | 16.00 (13.00, 21.00) | 16.00 (12.00, 22.00) | Z=-1.10 | 0.270 |
| Male age(y) | 31.00 (28.00, 34.00) | 31.00 (28.00, 34.00) | Z=-0.66 | 0.512 |
| Male BMI (kg/m2) | 25.71 (23.15, 28.39) | 25.43 (22.86, 28.09) | Z=-1.02 | 0.309 |
| Sperm concentration (10^6 /mL) | 51.23 (29.94, 79.84) | 51.06 (27.78, 81.72) | Z=-0.45 | 0.650 |
| Progressive motility (%) | 43.48 (34.20, 54.08) | 42.74 (32.31, 54.43) | Z=-1.01 | 0.313 |
| Sperm with normal morphology (%) | 3.50 (2.00, 5.00) | 3.50 (2.00, 5.00) | Z=-0.61 | 0.542 |
| DFI (%) | 11.84 (7.38, 18.01) | 11.90 (7.67, 17.80) | Z=-0.28 | 0.782 |
| Total Gn dose(IU) | 2606.25 (2125.00, 3231.25) | 2650.00 (2175.00, 3225.00) | Z=-0.26 | 0.795 |
| Duration of Gn stimulation(day) | 11.00 (10.00, 12.00) | 11.00 (10.00, 12.00) | Z=-0.37 | 0.709 |
| Estradiol, hCG day(ng/ml) | 2553.00 (1854.50, 3682.50) | 2606.00 (1789.50, 3688.00) | Z=-0.35 | 0.724 |
| Progestin, hCG day(ng/ml) | 0.71 (0.53, 0.91) | 0.71 (0.53, 0.90) | Z=-0.86 | 0.387 |
| Number of oocytes retrieved(n) | 12.00 (9.00, 16.00) | 12.00 (9.00, 16.00) | Z=-1.30 | 0.195 |
| ICSI rate (%) | 169 (22.01) | 419 (23.46) | χ²=0.64 | 0.423 |
| Group, n (%) |  |  | χ²=0.12 | 0.728 |
| Without blastocyst on Day 4 | 429 (55.86) | 986 (55.11) |  |  |
| With blastocyst on Day 4 | 339 (44.14) | 803 (44.89) |  |  |
| Data are M (Q₁, Q₃) or n. | | | | |
| BMI, body mass index. PCOS, polycystic ovary syndrome. FSH, follicle-stimulating hormone. LH, luteinizing hormone. AMH, anti-mullerian hormone. AFC, antral follicle count. DFI, DNA fragmentation index. ICSI, intracytoplasmic sperm injection. Gn, Gonadotropin. hCG, Human Chorionic Gonadotropin. | | | | |
