# Supplementary material for: Development and validation of a predictive model for early blastocyst formation on day 4 post-fertilization
Source: Front Endocrinol (Lausanne). 2026 Apr 1;17:1752963. doi: 10.3389/fendo.2026.1752963 (PMC13079057; doi:10.3389/fendo.2026.1752963)
Supplement: Supplementary Table 2 — Comparison of pregnancy outcomes following single high-quality embryo transfer on different days. Data are M (Q1, Q3) or n. aStatistically significant compared with cleavage on day 3; bStatistically significant compared with full compaction on day4; cStatistically significant compared with early blastocyst on day4. [file Table2.docx]

| **Supplementary Table S2.** Comparison of Pregnancy Outcomes Following Single High-Quality Embryo Transfer on Different Days | | | | |
| --- | --- | --- | --- | --- |
| Variables | Cleavage on day 3 | Full compaction on day4 | Early blastocyst on day4 | Blastocyst on day5 |
| Cases, n | 51 | 168 | 567 | 139 |
| Endometrial thickness, mm | 11.00 (9.50,12.00) | 11.50 (10.00,13.00) | 11.05 (10.00,13.00) | 11.50 (10.00,13.00) |
| Clinical pregnancy rate (%) | 35.29(18/51) | 55.95(94/168)^a^ | 64.19(364/567)^ab^ | 52.51(73/139)^ac^ |
| Ongoing pregnancy rate | 29.41(15/51) | 47.61(80/168)^a^ | 54.85(311/567)^a^ | 44.60(62/139)^c^ |
| Early miscarriage rate | 16.66(3/18) | 14.89(14/94) | 14.56(53/364) | 15.06(11/73) |
| Data are M (Q₁, Q₃) or n. ^a^Statistically significant compared with cleavage on day 3; ^b^Statistically significant compared with full compaction on day4; ^c^Statistically significant compared with early blastocyst on day4 | | | | |
